# Fabrication of Stable Nanofiber Matrices for Tissue Engineering via Electrospinning of Bare Laser-Synthesized Au Nanoparticles in Solutions of High Molecular Weight Chitosan

**DOI:** 10.3390/nano9081058

**Published:** 2019-07-24

**Authors:** Viraj P. Nirwan, Ahmed Al-Kattan, Amir Fahmi, Andrei V. Kabashin

**Affiliations:** 1 Faculty of Technology and Bionics, Rhine-Waal University of Applied Science, Marie-Curie-Straβe 1, 47533 Kleve, Germany; 2 Aix Marseille University, CNRS, LP3 (UMR 7341), 13288 Marseille, France; 3 MEPhI, Institute of Engineering Physics for Biomedicine (PhysBio), 115409 Moscow, Russia

**Keywords:** electrospinning, laser ablation, nanofibers, chitosan, poly(ethylene oxide) (PEO), Au nanoparticles, neutralization

## Abstract

We report a methodology for the fabrication of neutralized chitosan-based nanofiber matrices decorated with bare Au nanoparticles, which demonstrate stable characteristics even after prolonged contact with a biological environment. The methodology consists of electrospinning of a mixture of bare (ligand-free) laser-synthesized Au nanoparticles (AuNPs) and solutions of chitosan/polyethylene oxide (ratio 1/3) containing chitosan of a relatively high molecular weight (200 kDa) and concentration of 3% (*w*/*v*). Our studies reveal a continuous morphology of hybrid nanofibers with the mean fiber diameter of 189 nm ± 86 nm, which demonstrate a high thermal stability. Finally, we describe a protocol for the neutralization of nanofibers, which enabled us to achieve their structural stability in phosphate-buffered saline (PBS) for more than six months, as confirmed by microscopy and FTIR measurements. The formed hybrid nanofibers exhibit unique physicochemical properties essential for the development of future tissue engineering platforms.

## 1. Introduction

The elaboration of artificial scaffold platforms, capable of replacing and/or repairing failing tissues or full organs, is still challenging for the scientific community [1,2,3,4]. Most efforts are devoted to the fabrication of nanostructured scaffolds, which could mimic mesoporous morphologies of a real extracellular matrix (ECM) and offer additional therapy and diagnostics (theranostics) modalities [5,6,7]. Similar to conventional techniques, such as solvent casting and particle leaching, electrospinning is now extensively explored to elaborate structured biocompatible and biodegradable nanofibrous scaffolds. Such a technique can offer a series of advantages over traditional methods, including (i) the possibility of working with a variety of materials, including synthetic and natural polymers and their composites and (ii) the capability of generating micro- to nano-scale nanofibers having sophisticated special 3D designs [8]. Moreover, electrospun nanofibers can be employed as matrices for the encapsulation of drugs, as well as the incorporation of biological materials (e.g., proteins, DNA, etc.) and nanofunctional elements [9,10,11,12,13].

Chitosan is one of the most extensively exploited naturally derived biopolymers, which is widely used to develop novel electrospun nanofibrous matrices for a variety of biomedical applications, including anti-microbial, antifungal, wound healing, drug, and gene delivery [14,15,16,17,18]. Chitosan is characterized by a good biocompatibility and biodegradability, while the presence of NH_2_ groups on a chitosan nanofiber surface make it suitable for the immobilization of enzymes and negatively charged proteins. Moreover, a highly reactive chitosan surface can offer additional opportunities for its functionalization with nano-engineered particles to improve physicochemical characteristics (e.g., electrical and mechanical properties) and enable biological (e.g., antibacterial) or other theranostic functionalities [5,6,7,8,9,10,11,12,13,14,15,16,17,18,19]. However, the spinnability of chitosan polymer is relatively poor due to the presence of hydrogen bonds between polysaccharide chains, leading to its high crystallinity and weak solubility in most solvents. Therefore, acid solutions, such as acetic acid, trifluoroacetic acid (TFA), and ionic liquids are typically employed to facilitate the dissolution of chitosan. Moreover, due to a relative shortness of the chitosan chain, a polymer-stretching effect during electrospinning process is ineffective, which results in the formation of discontinued jets. To ensure the jet continuity and uniformity, one has to electrospin chitosan together with poly(ethylene oxide) (PEO) polymer [20,21,22]. Here, low molecular weight chitosan (102 kDa) looks preferable to ensure its complete dissolution, but such a choice typically requires high concentration of PEO to avoid the formation of small chitosan chains in the solution: This leads to a low concentration of chitosan in the matrix. On the other hand, the use of chitosan having too high a molecular weight (>310 kDa) results in the formation of gel structures, which are not suitable for electrospinning [23]. The employment of chitosan having reasonably high molecular weight (~200 kDa) looks a good compromise, but such a choice requires the elaboration and optimization of the whole electrospinning procedure [24].

Various chitosan/PEO formulations have been tested at different ratios of chitosan/PEO to obtain homogenous electrospun nanofibers with different characteristics [18,23,24,25,26]. In particular, we recently elaborated a novel procedure to fabricate chitosan/PEO nanofibers containing a relatively high molecular weight chitosan (200 kDa) and we decorated them with laser-synthesized Si and Au nanoparticles (SiNPs and AuNPs) as functional additives [5,19]. This procedure was based on the co-spinning of chitosan/PEO formulations (1% *w*/*v* of chitosan, ratio 1:4), together with nanoparticles prepared by methods of laser ablation, as described in our previous studies [27,28,29,30,31,32,33,34,35,36]. The uniqueness of laser-synthesized nanoparticles consists in their bare (ligand-free) and uncontaminated surface, which can provide a series of advantages for applications, including cancer diagnoses and therapies under external stimuli [32,33,34,35], biofuel cells [31], and Surface Enhanced Raman Spectrometry (SERS) [36], etc. As we showed in References [5,19], the incorporation of functional additives, such as Au and Si nanoparticles, into the chitosan/PEO matrix can provide a series of advantages, including: (i) A decrease of fiber size, which promises the improvement of its surface reactivity; (ii) an improvement of thermal stability at a high temperature; and (iii) the possibility for enabling additional theranostic modalities based on unique properties of laser-synthesized nanomaterials [5]. However, the total concentration of chitosan in the structure of nanofibrous remained relatively low compared to the co-spinning agent (ratio 1:4), which reduced the efficiency of the chitosan matrix and could lead to an unacceptably high rate of dissolution of chitosan/PEO nanofibers in an aqueous environment due to the presence of NH_3_^+^ [23,37,38].

In this article, we report a further advancement of the electrospinning procedure in order to improve the properties of the formed AuNPs-decorated hybrid chitosan/PEO nanofibers. Here, we show the possibility for increasing concentration of 200 kDa chitosan up to 3% (*w*/*v*) using 10% (*v*/*v*) acetic acid with a chitosan/PEO ratio of 1:3 in the electrospinning solution, which renders possible the solution of the dissolubility problem. Moreover, we describe a simple protocol of neutralization of NH_3_^+^ in the alkaline/alcoholic solution of 5 M NaOH or 1 M K_2_CO_3_ to ensure the structural stability of nanofiber membranes in biological media. Novel formulations of chitosan/PEO functionalized with bare laser-synthesized AuNPs look very promising for tissue engineering applications.

## 2. Materials and Methods

### 2.1. Materials

A medium molecular weight chitosan (200 kDa) and a PEO powder with the molecular weight of 300 kDa were purchased from Sigma-Aldrich (Darmstadt, Germany). A target (1 cm × 1 cm) of high purity Au (99.999%) was purchased from GoodFellow (Cambridge, United kingdom) to fabricate bare AuNPs. Chitosan was solubilized using acetic acid (99–100%) purchased from SCS GmbH (Sigmarszell, Germany). For the neutralization stage, methanol (99.9%), ethanol (99.5%), and K_2_CO_3_ were purchased from Carl Roth (Karlsruhe, Germany), while NaOH was purchased from VWR International GmbH (Langenfeld, Germany).

### 2.2. Methods

#### 2.2.1. Laser-Ablative Synthesis of Bare AuNPs

Bare laser-synthesized AuNPs were prepared using methods of femtosecond ablation and fragmentation in deionized water [27,30,31,33]. Briefly, a gold target (99.99%, GoodFellow, Cambridge, United Kingdom) was placed at the bottom of the glass vessel filled with deionized water (18.2 MΩ cm). A 2.3 mm diameter beam from a Yb:KGW laser (Amplitude Systems, 1025 nm, 480 fs, 1 kHz) was focused with a 75 mm lens on the surface of a target. The target was moved constantly in the focusing plane with a speed of 0.5 mm/s, while keeping the same thickness of the liquid (1 cm) above the target. The concentration of AuNPs was determined by the calculation of the weight loss of the target during the ablation process.

#### 2.2.2. Preparation of Electrospinning Solutions

A total of 2 mL of medium molecular weight chitosan was dissolved at a concentration of 3% (*w*/*v*) using 10% (*v*/*v*) acetic acid. In a separate cuvette, we prepared 4 mL of PEO with a concentration of 8% (*w*/*v*). The solutions were then mixed in the ratio of 1:3, respectively. A concentrated AuNPs solution was added to the polymer solution and ultra-sonicated for 2 h, which ensured the removal of bubbles and a homogenous dispersion of nanoparticles. Concentration of bare laser ablated AuNPs was measured to be 0.15 g L^−1^. The nanoparticle solution was concentrated before the addition to the electrospinning solution by heating it at 30 °C in an oven for several hours. Such a procedure led to 3-decreasing of the solution volume. A total of 2 mL of the concentrated solution 0.45 g L^−1^ was used to functionalize the electrospinning polymer solutions. This sample was denoted as Ch-AuNPs. A second sample of nanofibers without AuNPs (Ch-0) was prepared as a reference for comparative studies. In this case, 2 mL of AuNP solutions were replaced by 2 mL of PEO. The procedure was carried out in such a way that the final concentration of the polymer was the same as in the main sample (1:3 final ratio by volume). All steps of the preparation protocol are presented in Table 1.

#### 2.2.3. Electrospinning Hybrid Multifunctional Nanofibers

The fabrication of nanofibers was done using specialized equipment purchased from IME medical electrospinning technologies. A climate-controlled setup was employed to ensure constant environmental conditions and fixed parameters during the process. An electrospinning setup contained a rotating cylinder set at 2300 rpm. Electrospinning solutions were transferred into a 5 mL syringe, which was connected to a programmable pump providing the constant feed rate of 0.3 mL h^−1^. The pump was connected via a Leur-Lock and PTFE (Polytetrafluoroethylene) tube with a blunt 0.8 mm-diameter needle at one end of a spinneret, where 14 kV was applied to the solution. The distance between the collector and spinneret was kept at 15 cm based on previous optimization studies. The collector had a negative voltage of −1 kV and was wrapped in aluminium foil to collect the fibers. Climate conditions were fixed for all experiments at 25 °C temperature and 50% of humidity.

### 2.3. Morphological and Chemical Analysis

A high-resolution transmission electron microscopy (HR-TEM) system (JEOL JEM 3010, Croissy Sur Seine, France) was used to characterize laser-synthesized AuNPs. Thanks to ImageJ software (National Institutes of Health (NIH), Bethesda, MA, USA), statistic measurements were performed on more than 200 AuNPs to determine diameter size distribution. A scanning electron microscope was used as a primary analysis method to verify if the electrospinning process led to the formation of nanofibers. Due to the limited charging effect on samples, it was unnecessary to proceed to the sputter coating step. Size distributions and morphology of fibers were characterized using scanning electron microscopy (SEM), and a subsequent treatment of images using ImageJ^®^ software. JSM-IT100 InTouchScope™ system (Tokyo, Japan) operating at 5−20 kV accelerating voltages was the main microscope used at lower magnifications. A DSM 982 Gemini Zeiss system (Marly le Roi, France) operating at accelerating voltage of 20 kV was used to produce higher magnification micrographs. Both SEM systems were coupled to energy dispersive X-ray analysis. Chemical characteristics were evaluated by Fourier transform infrared (FTIR) spectroscopy (Perkin−Elmer, Cambridge, MA, USA) equipped with a universal attenuated total reflection (ATR) sampling accessory. The measurement histogram was performed by analyzing 2 micrographs; one from center of the collector, where most of the fiber deposition takes place, and another from the edge of the collector. The frequency distribution, as obtained using integrating ImageJ results with the origin, ranged from 100 to 15,000.

#### 2.3.1. Thermal Analysis

Thermal properties of nanofibers were studied with the help of thermogravimetric analysis (TGA, Perkin−Elmer TGA 4000 system, Billerica, MA, USA) and differential scanning calorimetry (DSC, Perkin−Elmer DSC 8000 system, Billerica, MA, USA). A total of 7 mg and 5 mg of nanofibers were used for each TGA and DSC measurement, respectively. A TGA heating chamber was flushed with nitrogen at 20 mL min^−1^, preventing the combustion of the samples, and heated at the rate of 10 °C min^−1^. Similarly, DSC was carried out under a nitrogen environment with a constant flow rate of 21 mL min^−1^ and heating rate was kept at 5 °C min^−1^. The operation temperature was between 30 and 700 °C during TGA studies and between 30 and 180 °C during DSC analysis.

#### 2.3.2. Neutralization of Chitosan/PEO Nanofibers

A total of two protocols were investigated based on two solutions of 1M K_2_CO_3,_ dissolved in 15 mL of ethanol (70%), and 5M NaOH dissolved in 15 mL of methanol (70%). The nanofibers were immersed in the solutions for a total of 20 h at ambient conditions. The immersion process was repeated 3 times by changing solutions after every immersion step to ensure effective neutralization. The samples were then washed with distilled water and dried at room temperature for 24 h before SEM-EDX and FTIR analyses to assess their structural stability and chemical composition.

## 3. Results and Discussion

**Figure 1:**
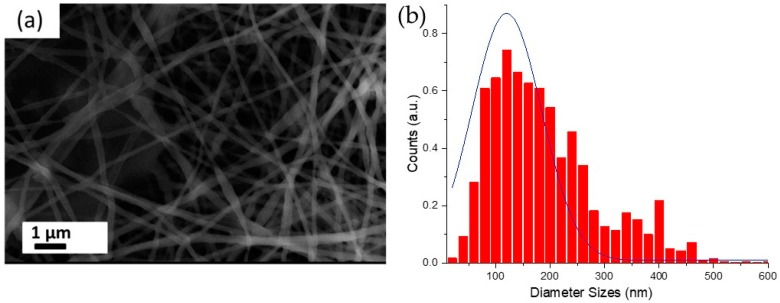
SEM image of hybrid chitosan/poly(ethylene oxide) (PEO) nanofibers (**a**) and corresponding size distribution for nanofiber thicknesses (**b**).

As mentioned above, the electrospinning of pure chitosan is quite challenging due to its poor solubility and short chain length. In most cases, 1% (*w*/*v*) concentration of chitosan is used at variable chitosan/PEO ratios. Recently, chitosan/PEO nanofibers functionalized with bare laser synthesized NPs were successfully electrospun [19]. In view to increase the available bioactivity of functional chitosan, new formulations have been tested. Based on our previous study, where we successfully fabricated chitosan/PEO nanofibers at a chitosan/PEO ratio of 1:4, the concentration of chitosan was increased from 1% (*w*/*v*) to 3% (*w*/*v*) and the chitosan/PEO ratio was increased from 1:4 to 1:3. Moreover, the concentration of acetic acid used to dissolve chitosan was also decreased down to 10% (*v*/*v*), as compared to highly acidic 90% (*v*/*v*) solutions used in our previous study [5,19]. Punctual structural analyses performed, thanks to SEM measurements, revealed that the nanofibers are homogenous without observable beads with a mean diameter of 189 nm ± 100 nm (Figure 1a,b).

**Figure 2:**
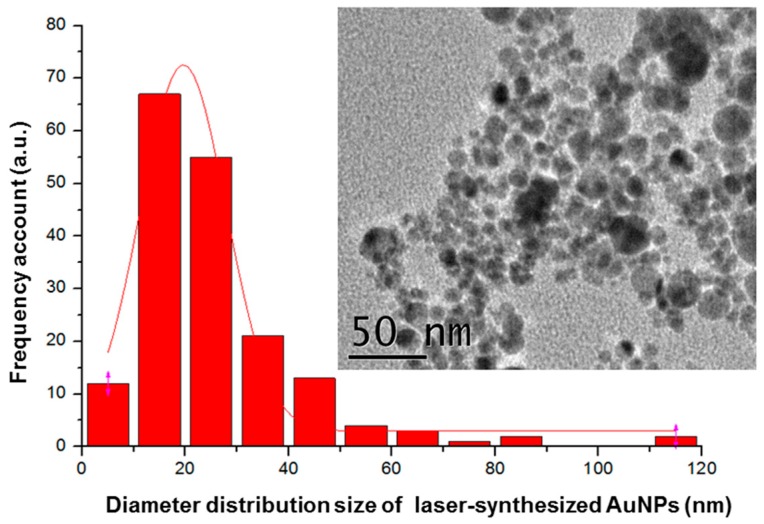
Typical high-resolution transmission electron microscopy (HR-TEM) image (inset image) of laser-synthesized AuNPs and corresponding nanoparticle size distribution.

Such initial composition of chitosan/PEO was thus used to prepare a novel solution containing highly concentrated AuNPs (0.09 mg mL^−1^ in electrospinning solution). A typical HR-TEM image of AuNPs formed by laser ablation and corresponding AuNPs size distribution is given in Figure 2.

**Figure 3:**
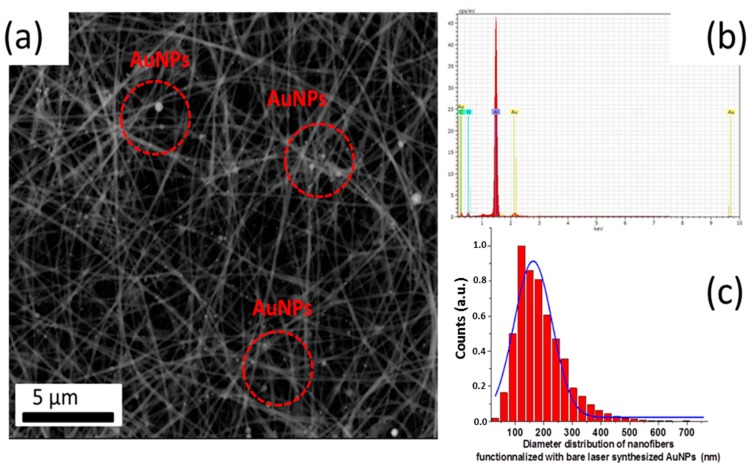
SEM image of hybrid chitosan/PEO nanofibers functionalized with bare laser-synthesized AuNPs (**a**); corresponding energy-dispersive X-ray spectrometry (EDX) spectrum (**b**); and size distribution of functionalized hybrid chitosan/PEO-AuNPs complex (**c**).

SEM-EDX examinations of chitosan/PEO nanofibers functionalized with AuNPs revealed a homogenous network of cylindrical nanofibers without any beads. All of these results are consistent with conclusions of our previous study [5], which evidenced similar morphologies of fibers electrospun with and without bare AuNPs. In addition, the fibers exhibited a mean diameter of 189 nm ± 86 nm, as measured on micrographs from different sample regions, using ImageJ^®^ software and fitted with a Gaussian approximation (blue curve) (Figure 3). AuNPs were clearly resolvable on the fiber surface, while the fiber matrix was still homogeneous. Energy-dispersive X-ray spectroscopy (EDX) confirmed the metallic nature of the bare laser AuNPs. The observed affinity between the nanofibers and the AuNPs was likely due to electrostatic interactions between the polycationic fiber surface with positively charged NH_3_^+^ groups and negatively charged (–23.1 ± 2.61 mV) Au nanoparticles [27,30,31,32,33]. It is worth noting that we recorded a 3.5-fold decrease of fiber diameter in comparison with chitosan/PEO nanofiber formulations studied in our previous work [5,19]. It is important that this result was obtained with 1:3 ratio of chitosan/PEO, promising higher reactivity of the nanofiber surface.

**Figure 4:**
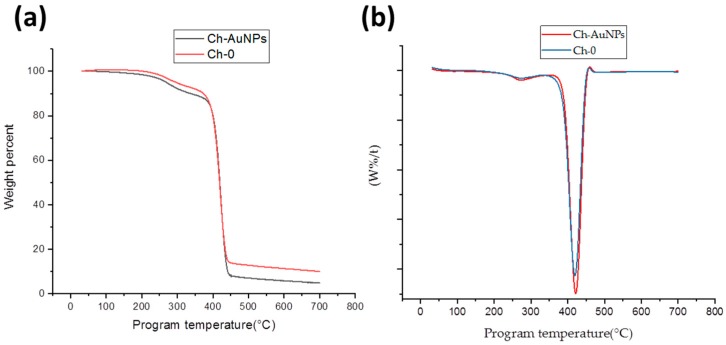
Thermogravimetric analysis (TGA) thermograms (**a**) with corresponding derivative thermogravimetric DTG curves (**b**) of chitosan/PEO nanofibers functionalized with bare laser AuNPs.

In our tests, we used TGA and DSC analyses to examine thermal properties of formed nanofibers and the impact of AuNPs on them. A TGA curve (Figure 4a) revealed that both Ch-0 and Ch-AuNPs exhibit the same behavior degradation. However, it is interesting to note that almost 10 weight % of the reference (Ch-0) is remaining after the heating cycle while 5 weight % of the functionalized nanofibers (Ch-AuNPs) was left after the program ended. This is likely due to the incorporation of AuNPs that act as heating spots leading to the maximum degradation of the polymer. The derivative curve of the TGA analysis was also provided in Figure 4b. When heated beyond their melting point, the nanofibers demonstrated a loss of weight. The weight loss region could be observed for both nanofibers functionalized with AuNPs (Ch-AuNPs) and no-functionalized nanofibers denoted as the reference sample (Ch-0) after 250 °C, which could be interpreted as the initialization of mass loss and could be attributed to the breaking of tertiary bonds in the polymer structure. The major weight loss region started at 347 °C and 366 °C for Ch-0 and Ch-AuNPs samples, respectively. The width of the peak evidenced a narrow range of temperatures, during which the maximum loss/degradation took place. Additionally, the peak temperature for functionalized nanofibers shifted up compared to the reference samples (from 418 to 423 °C).

**Figure 5:**
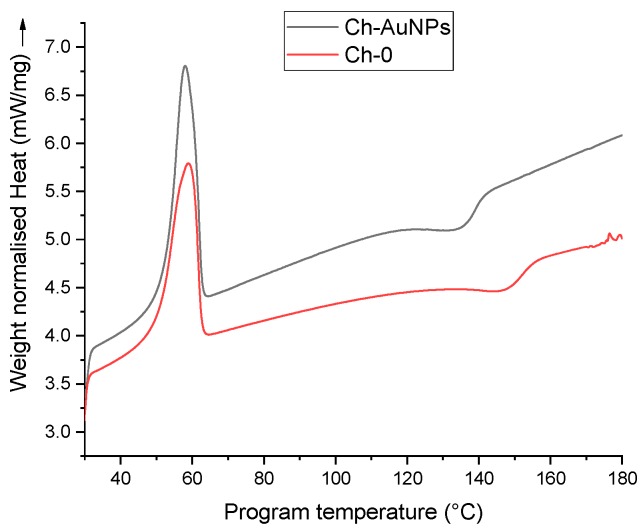
Differential scanning calorimetry (DSC) thermogram curve of chitosan/PEO reference (Ch-0) nanofibers and nanofibers functionalized with bare laser AuNPs (Ch-AuNPs).

Subsequently, DSC was carried out to record phase transition changes, which could take place e.g., at nanofiber melting points after their functionalization with gold nanoparticles. Here, both types of nanofibers displayed classical DSC curves. As shown in Figure 5, an endothermic peak could be observed for the reference nanofibers (Ch-0) at 59.10 °C, corresponding to the melting transition of PEO, while for Ch-AuNPs nanofibers, a similar peak was observed at 57.86 °C. Additionally, the normalized peak heat required to enable those transitions was 5.78 mW/mg and 6.81 mW/mg for Ch-0 and Ch-AuNPs, respectively. The calculated enthalpy for these samples provided 19.21 J/g and 16.96 J/g for Ch-AuNPs and Ch-0 samples, as measured by the analysis of the area under the curves. The nanoparticles used for functionalization absorbed additional heat and, therefore, more heat was required to enable a similar transition in functionalized nanofibers. Additionally, a second event was observed after the melting transition of nanofibers for both Ch-AuNPs and Ch-0. At 147.52 °C an exothermic peak could be observed for the reference sample and was at 134.90 °C for the functionalized nanofibers, which could be attributed to the Tg of the chitosan present in both samples. Therefore, it could be presumed that the heat provided while doing the analysis was not only limited by the nanofibers, but also by absorbing nanoparticles. Both TGA and DSC showed that the functionalization of nanofibers using bare laser AuNPs provided extra thermal stability in terms of absorbed heat and degradation rate. It should be noted that TGA and DSC data were highly reproducible based on the analysis of many samples.

**Figure 6:**
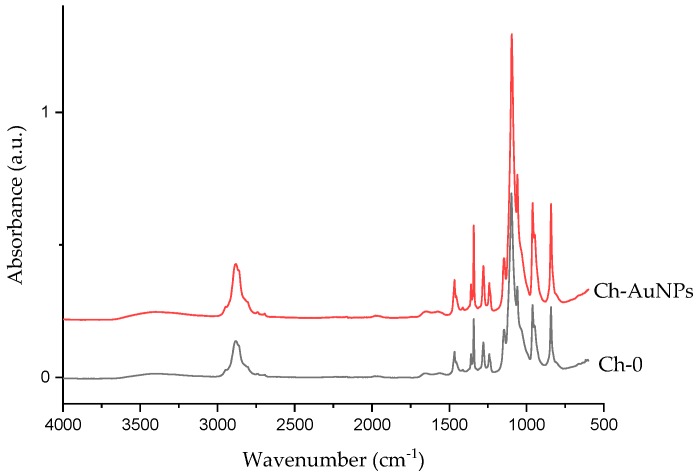
FTIR chitosan/PEO nanofibers functionalized with bare laser AuNPs.

The chemical composition of nanofibers was analyzed using FTIR before and after the functionalization. As shown in Figure 6, characteristic peaks could be observed for both constituent polymers (chitosan and PEO). The functionalization of nanofibers with bare laser AuNPs did not change chemical composition. Both the reference and the functionalized nanofibers displayed a broad band of NH_2_ and OH, stretching from 3500–3000 cm^−1^. A peak for amide absorption could also be observed at 1570 cm^−1^ for both functionalized and reference nanofibers [39,40,41]. A sharp and strong absorption peak attributed to CH_2_ stretching in PEO could also be observed at 2885 cm^−1^. The spectra beyond 1500 seems to be dominated with intense PEO bands at 1145, 1095, and 1059 cm^−1^, complimentary to C-O-C stretching vibrations [41,42]. The functionalization of nanofibers using bare laser AuNPs seems to have no impact on the chemical signature of nanofibers, as observed by FTIR

**Figure 7:**
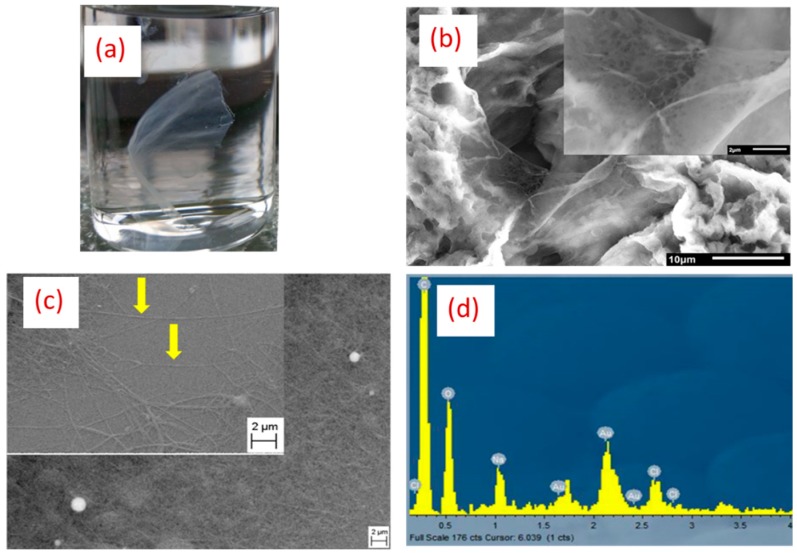
Macroscopic photo of chitosan/PEO functionalized with bare laser AuNPs neutralized with 1M of K_2_CO_3_ immersed in phosphate-buffered saline (PBS) for 24 h (**a**) with corresponding SEM images (**b**) (The inner image is given as illustration at higher magnification). SEM images of functionalized chitosan/PEO nanofibers with bare laser AuNPs neutralized with 5M NaOH (indicated by yellow arrows) in two different areas (**c**) with the corresponding EDX spectrum (**d**).

As mentioned in the introduction section, stability of chitosan in aqueous media is a crucial issue. High reactivity of the NH_3_^+^ group can lead to a rapid dissolution of chitosan/PEO complexes, making them hardly useful for applications in biological systems [28]. Hence, an additional neutralization step is needed to ensure the mechanical stability of the fibrous structure and its non-dissolvability in biological media. As mentioned earlier, two methods were tested to transform the NH_3_^+^ group into the NH_2_ insoluble form: (i) 1M K_2_CO_3_ dissolved in ethanol (70%) and (ii) 5M NaOH in methanol (70%). After the treatment, the samples were washed in distilled water and immersed in phosphate-buffered saline (PBS) for 24 h to test the neutralization efficacy. For the first method employing 1M K_2_CO_3_, the overall structure of the treated nanofibers had a non-woven mat-like morphology (Figure 7a).

**Figure 8:**
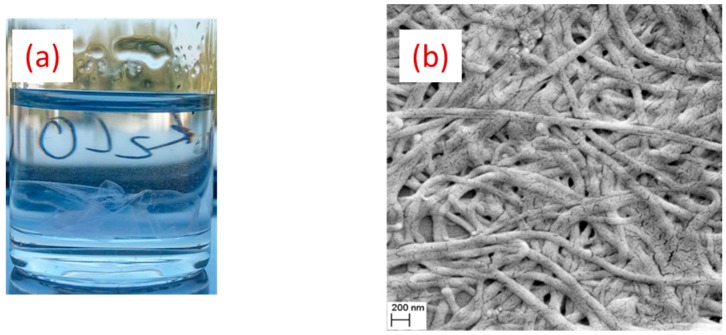
Macroscopic photo of functionalized chitosan/PEO with bare laser AuNPs immersed in PBS over six months (**a**) with the corresponding SEM image (**b**).

However, SEM examination of samples after their immersion in PBS solution for 24 h revealed the loss of the original fibrous structure, as one could not resolve individual nanofibers (Figure 7b), suggesting ineffective neutralization of NH_3_^+^. In contrast, when 5M NaOH solution in 70% methanol was used for neutralization, the overall nanofibers morphology was conserved at nano and macro scales, as it was indicted by the yellow arrows (Figure 7c). SEM analyses clearly showed that most of the fibers were present, while the presence of AuNPs could still be resolved by EDX analysis (Figure 7d). Moreover, we found the possibility for conserving the structural integrity of nanofibers over six months in PBS after neutralization with 5M NaOH. As shown in a macroscopic photo of nanofibers (Figure 8a,b), the immersion of nanofiber structures in biological PBS fluid did not lead to the dissolution of nanofibers or a dramatic change of their morphology. It is visible that some swelling and shrinkage effects could take place, but these structural changes do not look critical for projected applications, such as biomaterial matrices.

**Figure 9:**
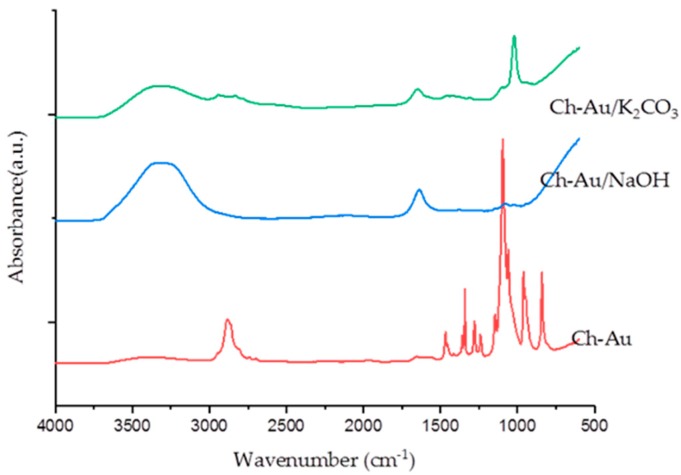
FTIR spectra of chitosan/PEO nanofibers functionalized with AuNPs before neutralization (Ch-Au) and after neutralization with K_2_CO_3_ (Ch-Au/K_2_CO_3_) or NaOH (Ch-Au/NaOH).

To confirm the conservation of chemical composition of chitosan/PEO nanofibers after their treatment by 1M of K_2_CO_3_ and 5M of NaOH, additional FTIR was carried out (Figure 9). Our analysis revealed that a large part of the PEO was removed, which was especially visible when 5M of NaOH was applied. In fact, main bands of pure PEO including 1467 cm^−1^, 1277 cm^−1^, 1343 cm^−1^, and 1283 cm^−1^ were not resolvable after such a treatment by NaOH, while a characteristic peak of chitosan amine at 3500 cm^−1^ became dominant. A secondary amine peak at 1680 cm^−1^ was also resolvable after both treatments, although this peak is often overshadowed by peaks from PEO [5,41]. Based on the first morphological assessment and FTIR studies, we can conclude that the neutralization of secondary amine in chitosan/PEO with 5M NaOH was successful. However, to make decisive conclusions on the efficiency of neutralization of AuNPs-decorated chitosan/PEO nanofibers and their potential for applications in biological systems, additional parameters (DSC, FTIR, TGA, size distribution, morphology) should be examined in detail. Results of such studies will be published elsewhere.

## 4. Conclusions

In summary, we prepared novel formulations of hybrid chitosan/PEO nanofibers, with the ratio of 1:3 at a relatively high concentration of chitosan 3% (*w*/*v*), and functionalized these nanofibers with bare laser-synthesized AuNPs. Thanks to systematic physicochemical analysis, improvements on the structural characteristics of the fibers were clearly demonstrated compared to previously reported nanofiber matrices [5,19]. First, microscopic measurements revealed a homogenous network of cylindrical fibers with AuNPs dispersed homogenously on the nanofibers surface without any effect on the chemical composition of the nanofibers, as shown by FTIR. Second, statistical measurements of the fiber size revealed a 3.5-fold decrease (189 ± 86 nm) fitted to a Gaussian monodispersion curve in comparison with the formulations of chitosan reported in our previous study (632 ± 169 nm) [5,19]. Thermal analysis showed that the incorporation of AuNPs into the chitosan/PEO nanofiber formulations improved their thermal stability. Finally, a simple neutralization protocol was proposed, which made possible the conservation of the structural integrity of nanofibers for a period of over six months. This work is a new encouraging step on the development of functional scaffold platforms based on hybrid chitosan/PEO nanofibers functionalized with bare nanoparticles.

## Figures and Tables

**Table 1:** Description of preparation steps for main and reference samples.

Main Sample Ch-AuNPs	Reference Sample Ch-0
3% (*w*/*v*) chitosan in 10% (*v*/*v*) acetic acid	3% (*w*/*v*) chitosan in 10% (*v*/*v*) acetic acid
8% (*w*/*v*) PEO in 2 mL conc. AuNPs in deionized water + 2mL deionized water	8% (*w*/*v*) PEO in 4 mL deionized water
1:3 final ratio by volume	1:3 final ratio by volume.
